# Complete and Durable Response to Nivolumab in Recurrent Poorly Differentiated Pancreatic Neuroendocrine Carcinoma with High Tumor Mutational Burden

**DOI:** 10.3390/curroncol28060388

**Published:** 2021-11-10

**Authors:** Nai-Wen Kang, Kien-Thiam Tan, Chien-Feng Li, Yu-Hsuan Kuo

**Affiliations:** 1 Division of Hematology and Oncology, Department of Internal Medicine, Chi-Mei Medical Center, Tainan 71004, Taiwan; wnk119@gmail.com; 2 ACT Genomics Co., Ltd., Taipei 114, Taiwan; jtchen@actgenomics.com; 3 Department of Medical Research, Chi-Mei Medical Center, Tainan 71004, Taiwan; angelo.p@yahoo.com.tw; 4 National Institute of Cancer Research, National Health Research Institutes, Tainan 70456, Taiwan; 5 College of Pharmacy and Science, Chia Nan University, Tainan 71710, Taiwan

**Keywords:** neuroendocrine carcinoma, immunotherapy, immune checkpoint inhibitor, tumor mutational burden

## Abstract

Poorly differentiated pancreatic neuroendocrine carcinomas (NECs) are rare and aggressive malignancies with rapid disease progression and early widespread metastasis. Given histology similarity, they are commonly treated with platinum-based chemotherapy as small cell lung cancer (SCLC). However, no standard treatment has been established for recurrent or progressive disease. We present an Asian patient with recurrent poorly differentiated pancreatic NEC after curative surgery and adjuvant chemotherapy with cisplatin and etoposide. The tumor mutational burden (TMB) was high. The patient received chemotherapy combined with maintenance immunotherapy with nivolumab and achieved promising and durable response, suggesting TMB could be a biomarker to identify NEC patients for immune checkpoint inhibitor (ICI) treatment.

## 1. Introduction

Pancreatic neuroendocrine carcinomas (NECs) are rare malignancies that develop from the endocrine tissues of the pancreas, accounting for less than 3% of primary pancreatic neoplasms [1]. They are poorly differentiated and characterized by aggressive natural history. Histologically, poorly differentiated NECs are similar to small cell lung cancer (SCLC) and thus treated based on studies for SCLC [2]. For localized disease, surgical resection followed by adjuvant chemotherapy with platinum drug plus etoposide is suggested [3]. However, there is no standard treatment established for recurrent or progressive disease. Several retrospective studies demonstrated that recurrent poorly differentiated NEC may benefit from subsequent-line chemotherapy [4,5,6]. The clinical outcome of poorly differentiated pancreatic NEC remains poor, with a median survival of 11 months [7].

Recent evidence has shown that immunotherapy with immune checkpoint inhibitors (ICIs) have antitumor activity against SCLC [8]. Results of phase 1/2 CheckMate 032 trial demonstrated antitumor activity and durable response in recurrent SCLC with nivolumab alone or combined with ipilimumab [9]. Phase 1b Keynote 028 trial indicated that pembrolizumab had promising antitumor activity in patients with previously treated PD-L1-expressing SCLC [10]. This study also demonstrated that pembrolizumab provided clinical meaningful antitumor activity in heavily pretreated carcinoids or pancreatic neuroendocrine tumors [11]. Here we present a case of recurrent poorly differentiated pancreatic NEC with high tumor mutational burden (TMB). The patient achieved complete and durable response with PD-1 inhibitor, nivolumab.

## 2. Case Report

The 59-year-old Asian male patient has underlying diseases of hypertension, abdominal aortic aneurysm and type 2 diabetes mellitus with medical control. Initially, he suffered from intermittent abdominal dull pain and fullness for a few days. He denied other specific complaints such as nausea, vomiting, diarrhea, jaundice, chest pain, headache, body weight loss, fever, and chills. Physical examination revealed a soft abdomen without tenderness, hepatomegaly, nor splenomegaly. The patient had an Eastern Cooperative Oncology Group (ECOG) performance-status score of 1.

**Figure 1:**
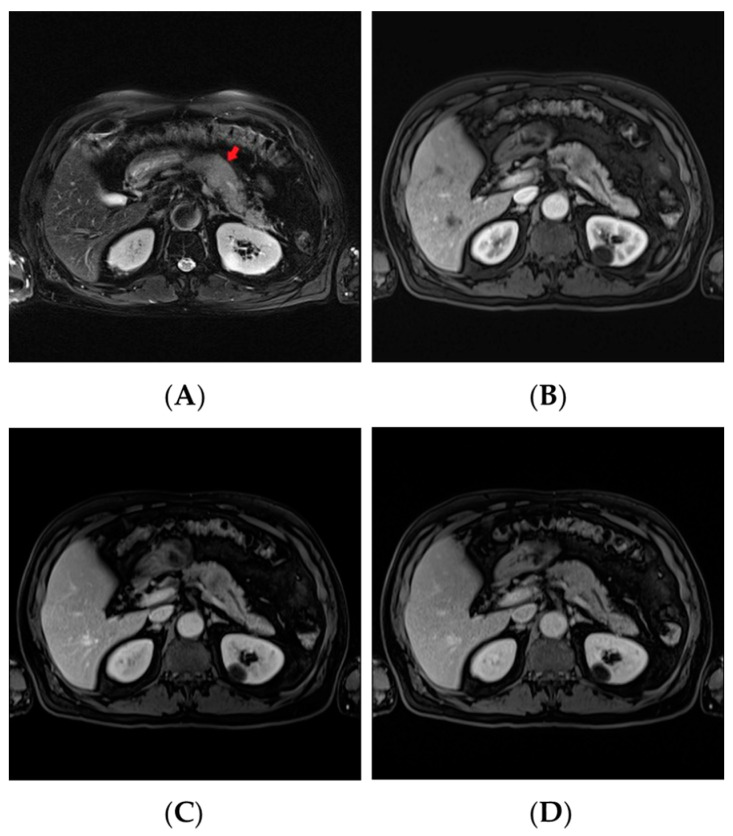
(**A**) Pancreatic magnetic resonance image (MRI) showed a pancreatic body hypovascular lesion (red arrow), approximately 3.3 cm, with peripancreatic invasion. (**B**) Arterial phase. (**C**) Portal venous phase. (**D**) Delayed phase.

Peripheral blood test revealed normal white blood cell count (9020/uL), hemoglobin (13.3 g/dL), and platelet count (206 × 103/uL). Liver function and renal function tests were normal. However, lipase was elevated (378 U/L) (normal range 8–78 U/L). A pancreatic body bulging hypodense nodular lesion with peripancreatic fat-stranding was noted on the computed tomography (CT) of abdomen. Furthermore, pancreatic magnetic resonance image (MRI) showed a hypovascular lesion at the pancreatic body, approximately 3.3 cm, with peripancreatic invasion and peripancreatic lymphadenopathy (Figure 1).

**Figure 2:**
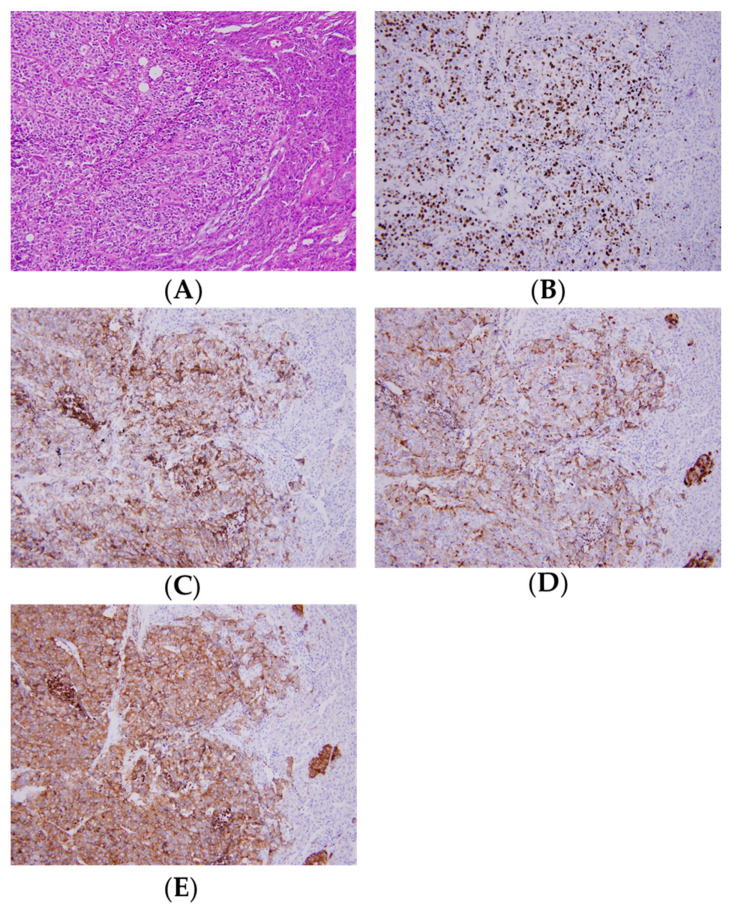
(**A**) Poorly differentiated neuroendocrine carcinoma (NEC) with more than 20 mitoses per 10 high power field. (**B**) The Ki-67 proliferation index was more than 20%. (**C**–**E**) The immunohistochemical (IHC) stain was positive for CD56, chromogranin A, and synaptophysin.

The patient underwent laparoscopic subtotal pancreatectomy. The pathology showed poorly differentiated small cell neuroendocrine carcinoma (NEC) with more than 20 mitoses per 10 high power field (Figure 2A). The Ki-67 proliferation index was 60% (Figure 2B). The immunohistochemical (IHC) stain was positive for neuroendocrine markers, including CD56, chromogranin A, and synaptophysin (Figure 2C–E). A total of 34 regional lymph nodes were resected, of which 2 were found to be involved. The patient was diagnosed as poorly differentiated pancreatic NEC with extension beyond the pancreas and regional lymph node metastasis. According to American Joint Committee on Cancer (AJCC) 8th, the pathological TNM staging was T2N1M0.

**Figure 3:**
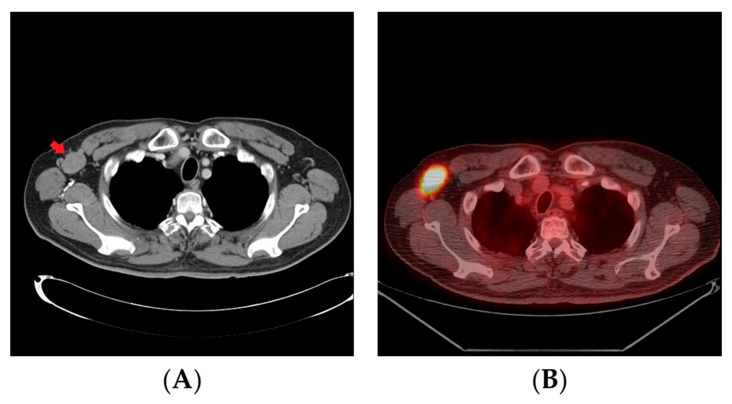
(**A**,**B**) Three months after the completion of adjuvant chemotherapy, the follow-up computed tomography (CT) scan and position emission tomography-computed tomography (PET-CT) revealed tumor recurrence at right axillary lymph nodes (red arrow).

After the surgery, the patient received six cycles of adjuvant chemotherapy with EP regimen (cisplatin 80 mg per square meter on day 1 and etoposide 80 mg per square meter from day 1 to day 3) every three weeks. However, three months after completion of adjuvant chemotherapy, the follow-up CT scan revealed tumor recurrence at right axillary and mediastinal lymph nodes (Figure 3A). The position emission tomography-computed tomography (PET-CT) showed multiple focal areas of increased F-18 fluorodeoxyglucose (FDG) avidity in right axilla, right lower para-tracheal region, bilateral pulmonary hila, and left para-abdominal aortic region (Figure 3B).

The tumor sections were stained immunohistochemically for programmed cell death-ligand 1 (PD-L1), which showed negative. After the pathology review, genomic DNA extracted from tumor and normal tissue was subjected to next-generation targeted sequencing (NGS) with a 440 cancer-related gene panel (ACTOnco^®^, Taipei, Taiwan, sequencing coverage 1.8 Mb; mean depth > 500×). After germline variant subtraction, thirty somatic mutations were identified, but none of them were actionable (Table 1). The tumor mutational burden (TMB) was 18.9 mutations/Mb, which is considered TMB-high. The microsatellite stability status determined by the NGS data was stable, and no deleterious mutations in the mismatched-repair genes were detected.

**Figure 4:**
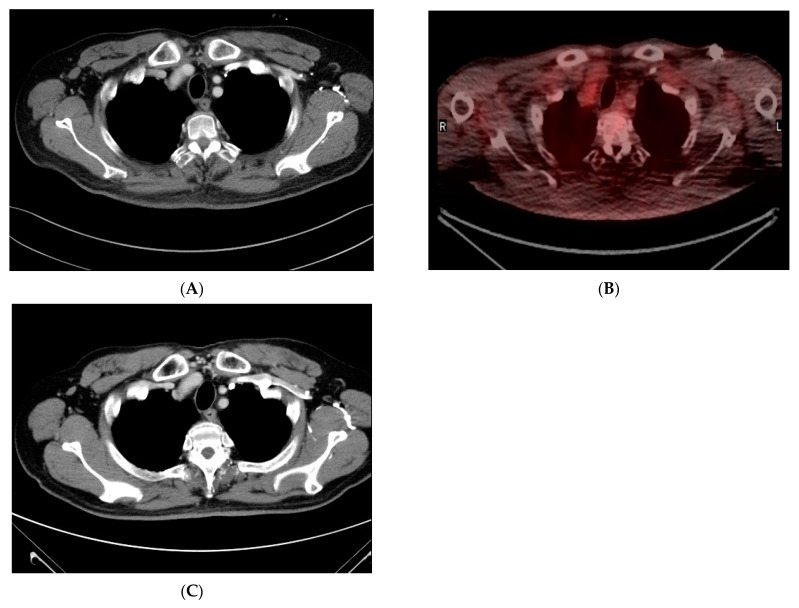
(**A**) After eight cycles of irinotecan and nivolumab, the follow-up CT scan showed a decrease in the size of the axillary lymph node and no residual mediastinal lymph nodes. (**B**) The follow-up PET-CT showed no F-18 fluorodeoxyglucose (FDG) avidity in right axilla, right lower para-tracheal region, bilateral hila, and left para-abdominal aortic region. (**C**) The patient had complete and durable response after four years of follow-up.

With the patient’s consent, a treatment regimen comprising irinotecan (135 mg per square) and immune checkpoint inhibitor nivolumab (200 mg) was administered every two weeks. After eight cycles of the combination therapy, the follow-up CT scan showed a decrease in the size of the right axillary lymph node and no residual mediastinal lymph nodes (Figure 4A). The follow-up PET-CT also showed no FDG avidity in the right axilla, right lower para-tracheal region, bilateral hila, and left para-abdominal aortic region (Figure 4B). We switched the regimen to maintenance nivolumab alone for two and a half years, which was discontinued due to grade 2 hepatitis. The patient remained disease-free at a follow-up of 4 years (Figure 4C).

## 3. Discussion

Neuroendocrine tumors (NETs) can arise in the endocrine system at different sites within the body, most commonly located in the gastrointestinal tract, pancreas, or lung. Pancreatic NETs are thought to develop from the endocrine tissues of the pancreas. The incidence of pancreatic NETs is low, accounting for less than 3% of primary pancreatic neoplasms [1]. NETs are rare among hypoenhancing pancreatic tumors, and must be discriminated from pancreatic adenocarcinomas [12]. NETs can be histologically classified based on tumor differentiation and grading, which is determined by mitotic count and Ki-67 proliferative index. Well-differentiated pancreatic NETs are divided into low grade (grade 1, Ki-67 index < 3%) and intermediate grade (grade 2, Ki-67 index 3–20%) [13]. High-grade (grade 3) pancreatic NETs are defined as Ki-67 index > 20%. Not all grade 3 tumors are poorly differentiated neuroendocrine carcinomas (NECs) and some are relatively well differentiated. Therefore, according to 2017 world health organization (WHO) classification, poorly differentiated NECs should be distinguished from well-differentiated grade 3 NETs [14].

We present a case of poorly differentiated pancreatic NEC, which is a rare and aggressive disease with rapid growth and high propensity of early metastasis. There is a lack of prospective studies guiding the treatment of poorly differentiated NECs. Given the similarities in morphology and biologic behavior to SCLC, poorly differentiate NECs are primarily treated based on SCLC studies [2]. For early-stage disease, combined multimodality treatment with surgery followed by adjuvant platinum-based chemotherapy with or without radiotherapy is recommended [3]. However, the majority of the patients eventually encounter tumor recurrence or progression after front-line chemotherapy. To date, there is limited data on subsequent therapy and no standard treatment has been established for recurrent poorly differentiated NEC. Some retrospective studies or case series demonstrated limited clinical benefit with subsequent-line chemotherapy, such as irinotecan, topotecan, and temozolomide [4,5,6]. However, the prognosis remains poor and effective novel therapies are desperately needed.

Recent studies have demonstrated that immunotherapy with immune checkpoint inhibitors (ICIs) have promising antitumor responses and survival benefits in a variety of malignancies, including melanoma, non-small cell lung cancer (NSCLC), and head and neck cancer [15,16,17,18,19]. The best-characterized ICIs are antibodies that target programmed cell death protein-1 (PD-1) (nivolumab and pembrolizumab), PD-L1 (atezolizumab) and cytotoxic T-lymphocyte antigen-4 (CTLA-4) (ipilimumab), the goal of which is to enhance the immune system for the detection and eradication of tumor cells. To date, clinical trials of immune checkpoint inhibitors have also been investigated in SCLC and shown antitumor activity. CheckMate 032, a phase 1/2 study, demonstrated that nivolumab alone or combined with ipilimumab generated antitumor activity and durable response in patients with recurrent SCLC [9]. In Keynote 028, a phase 1b trial, pembrolizumab had promising antitumor activity in patients with previously treated PD-L1-expressing SCLC [10]. Moreover, this study demonstrated that pembrolizumab provided clinical meaningful antitumor activity in heavily pretreated carcinoids or pancreatic NETs [11].

Some case reports of ICIs for NECs have been published. Paraghamian et al. reported a patient with recurrent metastatic small cell NEC of the cervix who achieved a complete response to nivolumab [20]. Wang et al. presented a case of large cell NEC of the lung progressing after surgery and adjuvant chemotherapy who had a durable response with pembrolizumab [21]. We reported a case of recurrent poorly differentiated pancreatic NEC treated with chemotherapy plus ICI, nivolumab. The prognosis of poorly differentiated pancreatic NEC is poor and most patients die within less than a year [7]. In our case, the patient achieved sustained response for 4 years. ICIs can be considered as effective therapeutic agents for poorly differentiated NEC.

Nivolumab is a fully human IgG4 monoclonal antibody that targets the PD-1 receptor on T cells. Given that the PD-1/PD-L1 pathway in the tumor microenvironment may induce suppression of the immune system, PD-L1 expression has been investigated as a predictive biomarker for anti-PD1/PD-L1 therapy. PD-L1 expression can be detected in poorly differentiated NECs, approximately 14% on tumor cells and 27% on tumor-associated immune cells [22]. In addition, PD-L1 expression is significantly associated with high-grade NETs [23]. However, PD-L1 expression in our case was negative. Some evidence showed that PD-L1 expression is associated with higher response rates and prolonged survival with anti-PD-1/PD-L1 therapy [16,17,18]. Actually, tumor response to anti-PD-1/PD-L1 therapy can be observed in PD-L1-negative tumors and not all PD-L1-positive tumors respond to anti-PD-1/PD-L1 therapy. Paraghamian et al. and Wang et al. also presented cases of poorly differentiated NECs with absent PD-L1 expression that achieved promising response to anti-PD1 monoclonal antibody [21]. Both of the cases reported by Paraghamian et al. and Wang et al. had high mutation burden, which is considered as a promising predictive marker of ICIs.

**Figure A1:**
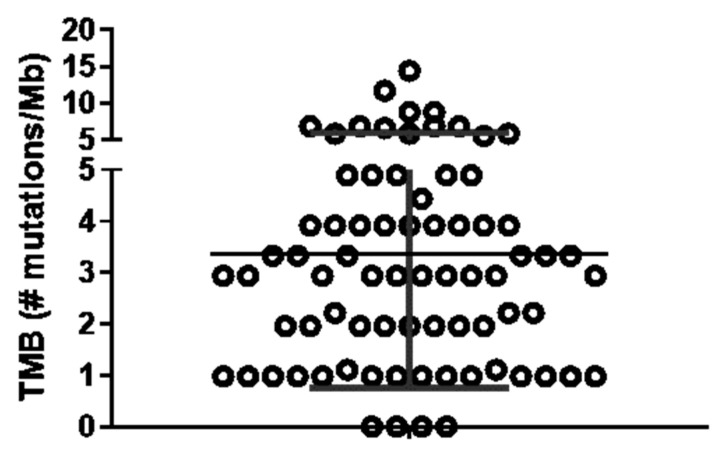
Distribution of tumor mutational burden of 75 pancreatic neuroendocrine tumors derived by MSK-IMPACT panel.

The patient’s tumor harbors 35 mutations/Mb and is classified as high tumor mutational burden (TMB). The predictive value of TMB to anti-PD1/PD-L1 and anti-CTLA4 therapies has been shown across multiple cancer types, including melanoma and non-small cell lung cancer (NSCLC) [24,25], which led to the US Food and Drug Administration (FDA) approval of TMB-high as a tumor agnostic predictive biomarker for pembrolizumab in 2020. Increasing evidence suggested that cut-off for TMB-high may vary significantly between histologies, and a universal TMB threshold may not be appropriate. A retrospective TMB analysis carried out by Memorial Sloan Kettering Cancer Center (MSKCC) showed that patients with a tumor harboring top quantile (highest 20%) TMB within the same histology tended to have improved overall survival [26]. We further analyzed 75 pancreatic neuroendocrine tumors sequenced by the MSK-IMPACT panel of MSKCC and found the highest 20% TMB being 4.9 mutations/Mb (Figure A1).

Based on the analysis of the genetic alteration profile of the tumor, there were no preexisting genomic alterations in antigen processing and presentation as well as immune response, such as B2M [27,28], PTEN [29,30], STK11 [31,32,33,34], which might account for *de novo* resistance to ICI treatment. Somatic mutations in SERPINB3, a gene encoding a protein of the serpin family of serine protease inhibitors, were reported to predict improved survival from treatment with anti-CTLA4 therapy in two independent cohorts of patients with melanoma [35].

Besides interfering with deoxyribonucleic acid (DNA) synthesis and replication, conventional cytotoxic chemotherapy may stimulate the immune system through several modalities and induce an immunogenic cell death in tumor cells [36]. In addition, some cytotoxic agents increase the ratio of cytotoxic lymphocyte to regulatory T cells [37]. The potential immunogenic effects of chemotherapy may help modulate immune response through PD-l/PD-L1 inhibitor. These studies provide the rationale for the combination of immunotherapy and chemotherapy to enhance antitumor activity and achieve better clinical outcome. A phase 3 IMpower133 study has demonstrated that the addition of atezolizumab to chemotherapy resulted in significant longer overall survival and progression-free survival than chemotherapy alone in patients with previously untreated extensive-stage SCLC [38]. In our case, we administered immune checkpoint inhibitor (ICI) combined with cytotoxic chemotherapy, which may induce immunogenic effects and trigger the antitumor activity of ICI. In terms of recurrent or progressive poorly differentiated NECs, adding ICI to chemotherapy may be a promising strategy. Further analysis of tumor mutational burden can provide clinicians with more information to evaluate the use of ICI.

## 4. Conclusions

Poorly differentiated pancreatic neuroendocrine tumors (NECs) are rare and aggressive malignancies with poor prognosis. There are currently no consensuses on the standard treatment for the recurrent or progressive disease after failing platinum-based chemotherapy. Other than conventional cytotoxic agents, immune checkpoint inhibitors (ICIs) targeting the PD-1/PD-L1 pathway may achieve promising and durable response in patients with recurrent pancreatic NECs. Tumor mutational burden (TMB) could be a biomarker to evaluate the efficacy of ICIs in poorly differentiated pancreatic NECs. More research is needed to search the predictive biomarkers of ICIs, which may have clinical benefit in a specific subset of patients with poorly differentiated pancreatic NECs.

## Figures and Tables

**Table 1:** Thirty somatic mutations identified by the next-generation targeted sequencing with a 440 cancer-related gene panel.

Gene	Chr	Exon	Accession Number	cDNA Change	Amino Acid Change	Coverage	Variant Allele Frequency (VAF)	COSMIC ID
*ADAMTS6*	5	15	NM_197941	c.1861G > T	p.Glu621Ter	463	18.9%	-
*ADAMTSL1*	9	-	NM_001040272	c.4643 + 1G > T	splice donor	1110	26.8%	-
*AMER1*	X	2	NM_152424	c.2265G > T	p.Glu755Asp	422	63.8%	-
*AXIN1*	16	-	NM_003502	c.1020-5G > T	splice region	1435	29.2%	-
*AXL*	19	20	NM_021913	c.2446C > A	p.Pro816Thr	788	39.6%	-
*BCL9*	1	8	NM_004326	c.2151G > T	p.Lys717Asn	298	19.1%	-
*BRD4*	19	10	NM_058243	c.1895G > A	p.Arg632His	1497	56.7%	COSM4666105
*CDC73*	1	3	NM_024529	c.306G > A	p.Ala102=	436	33.0%	COSM4026318
*DICER1*	14	11	NM_177438	c.1808C > A	p.Pro603His	586	60.9%	-
*DNMT3A*	2	14	NM_175629	c.1607A > G	p.Tyr536Cys	705	37.1%	-
*DNMT3A*	2	-	NM_175629	c.639 + 8G > A	splice region	1394	16.8%	-
*EP300*	22	31	NM_001429	c.7223A > T	p.Gln2408Leu	533	45.5%	-
*EPHA7*	6	-	NM_004440	c.2173-1delG	splice acceptor	515	16.3%	-
*ERBB2*	17	27	NM_004448	c.3616C > T	p.Gln1206Ter	564	16.8%	-
*FGFR4*	5	3	NM_213647	c.184C > A	p.Arg62Ser	2221	19.8%	-
*FGFR4*	5	17	NM_213647	c.2158G > T	p.Gly720Trp	755	29.4%	-
*HR*	8	-	NM_005144	c.2367 + 3G > C	splice region	505	22.9%	-
*KMT2A*	11	-	NM_001197104	c.5364-3C > T	splice region	802	18.0%	-
*KMT2D*	12	11	NM_003482	c.3308G > T	p.Cys1103Phe	1128	32.0%	-
*LRP1B*	2	89	NM_018557	c.13516G > A	p.Asp4506Asn	471	19.1%	COSM3567099
*LRP1B*	2	67	NM_018557	c.10470dupC	p.Asp3491ArgfsTer6	484	41.1%	-
*MAX*	14	4	NM_002382	c.172-1_172delGGinsTT	splice acceptor	625	52.6%	-
*MUC16*	19	14	NM_024690	c.36746G > T	p.Arg12249Leu	2158	57.9%	-
*NSD1*	5	23	NM_022455	c.6611A > C	p.Glu2204Ala	876	20.7%	-
*PTPRD*	9	38	NM_002839	c.5048C > A	p.Ser1683Tyr	625	19.9%	COSM6961014
*PTPRD*	9	-	NM_002839	c.2350-1G > T	splice acceptor	645	19.4%	-
*PTPRT*	20	16	NM_007050	c.2435C > A	p.Thr812Asn	1873	28.0%	-
*PTPRT*	20	12	NM_007050	c.1948G > T	p.Val650Leu	2553	41.5%	-
*SERPINB3*	18	8	NM_006919	c.1061C > A	p.Ser354Ter	965	30.6%	COSM6149208
*STAG2*	X	8	NM_001042751	c.568A > G	p.Ile190Val	276	64.0%	-
*TERT*	5	9	NM_198253	c.2476G > A	p.Val826Ile	1040	21.8%	COSM6916168
*TET1*	10	2	NM_030625	c.981A > G	p.Ile327Met	1780	30.1%	-
*TP53*	17	7	NM_000546	c.774A > T	p.Glu258Asp	1587	35.7%	COSM44962
*TP53*	17	5	NM_000546	c.422G > A	p.Cys141Tyr	1021	30.1%	COSM43708
*TSC2*	16	12	NM_000548	c.1171G > A	p.Val391Met	983	36.6%	-

## Data Availability

Data sharing not applicable.
